# A Metalated Porous Porphyrin Polymer with [Co(CO)_4_]^−^ Anion as an Efficient Heterogeneous Catalyst for Ring Expanding Carbonylation

**DOI:** 10.1038/s41598-018-31475-6

**Published:** 2018-09-05

**Authors:** Jianwei Jiang, Sungho Yoon

**Affiliations:** Department of Applied Chemistry, Kookmin University, 861-1, Jeongneung-dong, Seongbuk-gu, Seoul, 02707 Korea

## Abstract

The synthesis of β-lactones from epoxides through ring-expanding carbonylation using homogeneous catalysts has received much attention. However, homogeneous catalysts suffer from difficulty in product separation and recycling of the catalyst, limiting their industrial usage. Herein, a novel heterogeneous catalyst, [Cr-metalated porous porphyrin polymer]^+^[Co(CO)_4_]^−^, was prepared and used for the conversion of propylene oxide (PO) to β-butyrolactone; this catalyst presented superior catalytic activity and selectivity (99%) than our previous heterogeneous catalyst. In addition, the catalyst was readily separated from the product without significant loss of catalytic activity. A possible method to recover the original catalytic activity also was demonstrated.

## Introduction

β-lactones are important intermediates in organic and polymer chemistry^1–3^. In particular, β-butyrolactone can be used to synthesize poly (3-hydroxybutyrate), which is a class of potential thermoplastic biopolymers^4–6^. However, the synthesis of β-lactones is challenging, resulting in their under utilization in industry^7–11^.

**Figure 1:**
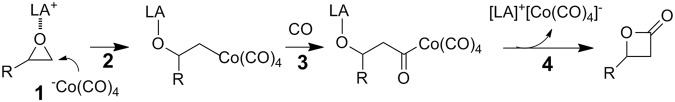
Schematic depiction of proposed mechanism for epoxide carbonylation by [Lewis acid]^+^[Co(CO)_4_]^−^.

As one solution to this challenge, the ring-expanding carbonylation of epoxides has been suggested, not only because of the cheap epoxide and carbon monoxide, but also because of the efficient one-step procedure^9–15^. Recently, this strategy has been demonstrated to be viable with the development of the bimetallic catalyst [Lewis acid]^+^[Co(CO)_4_]^−^ in epoxide carbonylation^10–21^. Notably, Coates’ group developed a class of well-defined catalysts, such as [(porphyrin) Cr or Al]^+^[Co(CO)_4_]^−^ and [(salph) Cr or Al]^+^[Co(CO)_4_]^−^, for epoxide carbonylation^10–13,19–21^. Additionally, the mechanism for epoxide carbonylation by [Lewis acid]^+^[Co(CO)_4_]^−^ was proposed: (1) epoxide activation by the [Lewis acid]^+^, (2) attack by Co(CO)_4_^−^ to form a ring-opened intermediate, (3) CO insertion into Co-alkyl bond, and (4) ring-closure, which results in β-lactone formation (Fig. 1)^22,23^. Although these catalysts are highly efficient, their application is limited because of the difficulties associated with their separation and reuse.

To overcome these drawbacks, a heterogeneous catalyst [bpy-CTF-Al(OTf)_2_]^+^[Co(CO)_4_]^−^ was first synthesized by us for epoxide carbonylation to lactone^24^. However, this catalyst possesses limited catalytic activity (site time yield (STY) value 1.1 h^−1^) and selectivity (90%). Interestingly, Roman-Leshkov’s group recently exploited another heterogeneous catalyst, Co(CO)_4_^−^-incorporated Cr-MIL-101, for epoxide carbonylation; the STY value (176 h^−1^, 1,2-epoxyhexane) was comparable to that of various homogeneous catalysts^15^. However, its activity was far lower than that of the optimized homogeneous catalyst (STY 740 h^−1^, 1,2-epoxyhexane)^20^. Therefore, the development of a heterogeneous catalyst for further improvement of catalytic performance for epoxide carbonylation is highly desirable and of great importance.

Generally, the Cr-porphyrin homogeneous catalysts present superior catalytic performance compared with other catalysts^10–13,19–21^. Therefore, [Cr-metalated porous porphyrin polymer]^+^[Co(CO)_4_]^−^ heterogeneous catalyst was synthesized and used for PO carbonylation. In addition, facile separation and successive recycling of the catalyst are presented.

## Results and Discussion

**Figure 2:**
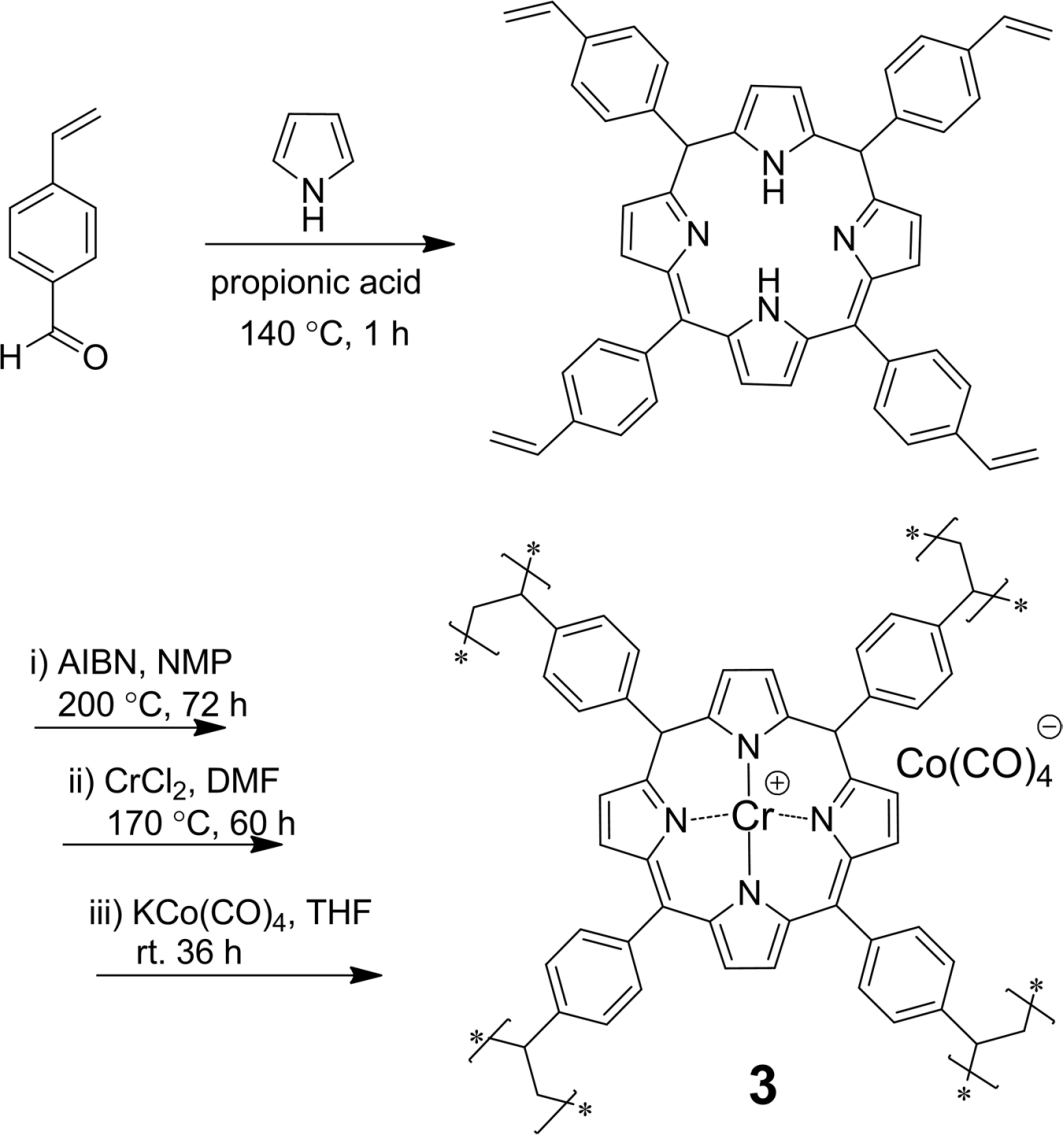
Schematic depiction for the synthesis of catalyst **3**.

The porphyrin-based porous organic polymer (**1**) was synthesized according to a recently published method developed by Xiao’s group (Fig. 2 and Supplementary Information)^25^. An scanning electron microscopy (SEM) image of **1** showed irregular particles, and the individual particles were composed of aggregated ~15-nm primary nanoparticle (Supplementary Fig. S2). The porous structure of the material was confirmed using N_2_ sorption isotherms at 77 K. The average pore diameter for polymer **1** is 4.8 nm (Supplementary Fig. S3). The Brunauer-Emmett- Teller (BET) surface area of **1** is 1109 m^2^/g, with a total pore volume of 1.3 cm^3^/g (Fig. 1b). These data are similar to previously reported results (1200 m^2^/g, 1.3 cm^3^/g), indicating that porous polymer **1** with a high surface area was well synthesized. An amorphous peak in the 14° to 28° range was detected in the powder X-ray diffraction (XRD) pattern, suggesting this material is amorphous (Supplementary Fig. S4).

**Figure 3:**
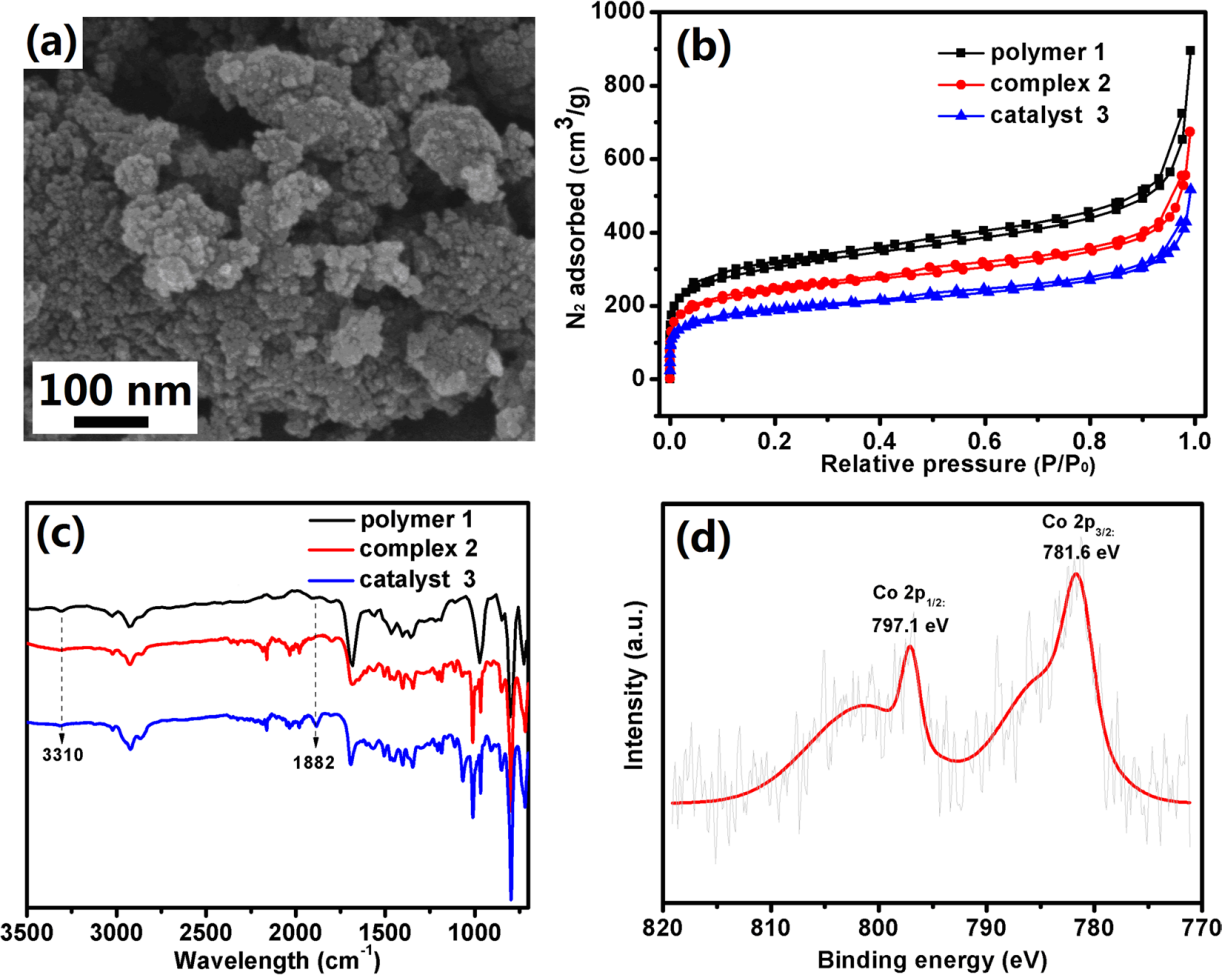
(**a**) SEM image of catalyst **3** (**b**) BET, and (**c**) IR of polymer **1**, complex **2**, and catalyst **3** (**d**) XPS of Co(2p) peaks of catalyst **3**.

Then, synthesized polymer **1** was metalated according to the synthetic procedure for homogeneous Cr(III) metalporphyrin complexes (Fig. 2)^26^. Specifically, polymer **1** was treated with CrCl_2_ in dimethylformamide at 170 °C for 1 h to give a powder (yield, 93%). An obvious color difference between these materials before (reddish brown) and after (light reddish) metalation was observed. Infrared (IR) measurements showed the intensity of the N-H stretch at 3318 cm^−1^ was reduced significantly upon metalation of polymer **1**, suggesting that Cr(III) ions were inserted into the porphyrin cores and substituted for its two protons (Fig. 3c). Energy-dispersive X-ray (EDX) analysis showed that only ~20.0% of porphyrin units in polymer **1** were coordinated to metal atoms, based on the calculation of the Cr/N atomic ratio (Supplementary Fig. S5). By extending the reaction time to 60 h, the content of metalated porphyrin polymer (complex **2**) was improved to 55.5%, as determined by EDX (Supplementary Fig. S6). Inductively coupled plasma atomic emission spectroscopy (ICP-AES) revealed that the ratio of metalated porphyrin in complex **2** is 58.9%, which is close to the value determined by EDX analysis. The reason for the partial metalation may be ascribed to the poor solubility of highly cross-linked polymer **1**, resulting in fewer porphyrin sites available for metalation.

To replace Cl^−^ in complex **2** (58.9% metalated porphyrin) with Co(CO)_4_^−^, complex **2** was treated with excess KCo(CO)_4_ to give catalyst **3** with excellent yield (96%). An SEM image of catalyst **3** demonstrated that the nanoparticle-aggregated hierarchical structure was maintained after the reaction (Fig. 3a). The color difference between complex **2** (dark) and catalyst **3** (dark brown) suggested the anion exchange may occur (Supplementary Fig. S7). For catalyst **3**, the IR absorption peak at 1882 cm^−1^ is the characteristic absorption of the CO in Co(CO)_4_^−^ (Fig. 3c)^10,19–21,24^. In the X-ray photoelectron spectroscopy (XPS) spectrum, the Cr(2p_1/2_) electron binding energy was found to be 586.5 eV, which is 0.7 eV lower than that of complex **2** (Supplementary Fig. S8). The difference of Cr(2p_1/2_) binding energy may be due to the anion exchange between Cl^−^ and Co(CO)_4_^−^. After the catalyst **3** was purified several times using the process of dispersion/filtration/washing with THF, a few of potassium species (0.17 wt%) was detected from the EDX analysis (Supplementary Fig. S9), due to the poor solubility of KCl in THF.

To identify the exact amount of Co in catalyst **3**, ICP was used, showing that the Co and Cr contents were 1.9 wt% and 3.4 wt%, respectively; the atomic ratio of Cr/Co was 2:1, which is in agreement with the EDX analysis (Cr/Co: 2.1:1, Supplementary Fig. S9). These results indicate that only half of the Cr species was paired with Co(CO)_4_^−^. The other half of the Cr species may exist as ion pairs with Cl^−^ as a counter ion.

XPS demonstrated the presence of the Co anion in catalyst **3**, corresponding to the Co^−^ (2p_3/2_) peak at 781.6 eV and Co^−^ (2p_1/2_) peak at 797.1 eV, which is in agreement with our previous reports^24,27^. This result further demonstrated the successful anion exchange (Fig. 3d).

The reactivity of porous material-supported catalysts depends on the porosity and pore size of the support. To characterize the porosity of complex **2** and catalyst **3**, BET analysis was performed. The nitrogen sorption isotherms of these two materials showed the combined features of type I and type IV curves (Fig. 3b). Specifically, the rapid adsorption in the P/P_0_ <0.1 range is due to the filling of micropores (type I); the obvious hysteresis loop and a sharp upturn at high relative pressure (P/P_0_ = 0.9) indicate the presence of mesopores and macropores in these materials (type IV). The average pore diameters for complex **2** and catalyst **3** are 4.7 nm and 4.6 nm, respectively. The BET surface areas of complex **2** and catalyst **3** are 877 and 676 m^2^ g^−1^, with pore volumes of 1.0 and 0.8 cm^3^ g^−1^, respectively. These results may be attributed to the ion exchange between Cl^−^ and Co(CO)_4_^−^, resulting in the immobilization of Co(CO)_4_^−^ within the porous polymer. Notably, the pore size in catalyst **3** is large enough to allow the diffusion of reactants, including CO, PO, and β-butyrolactone.

The catalytic performance of catalyst **3** was examined using carbonylation of PO. First, PO carbonylation was performed in a 100-mL stainless steel tube reactor with homogeneous [(TPP)Cr(THF)_2_]^+^[Co(CO)_4_]^−^ catalyst (1 mol% to PO) in THF at 60 °C under 60 bar of CO for 3 h. The crude product of PO carbonylation was analyzed by ^1^H NMR spectroscopy with naphthalene as an internal standard. PO was converted to β-butyrolactone in 94% yield and ignorable acetone (<1%) (Table 1-1). Next, the activity of catalyst **3** was evaluated under the same reaction conditions. To our delight, the β-butyrolactone was obtained in 93% yield and the molar ratio of β-butyrolactone to acetone was 98:2, which was close to the result for the homogeneous catalyst (Table 1-2). The obtained STY value (31 h^−1^) was higher than that of our heterogeneous catalyst [bpy-CTF-Al(OTf)_2_]^+^[Co(CO)_4_]^−^ (1.1 h^−1^, Table 1-6) but lower than Roman-Leshkov’s catalyst Co(CO)_4_^–^incorporated Cr-MIL−101 (STY 176 h^−1^, Table 1-7). Importantly, this STY value was comparable to that of various homogeneous catalysts used in PO carbonylation (Table 1, 8–11).

**Table 1:** Catalysts for epoxide carbonylation.

No	Catalyst	Epo.	Sol.	T (°C)	P_CO_ (bar)	t (h)	Epoxide/Co^a^	Yield (%)^b^	Lactone: ketone^b^	STY (h^−1^)^c^	Ref
1	[TPPCr(THF)_2_]^+^ [Co(CO)_4_]^−^	PO	THF	60	60	3	100	94	>99:1	31	this work
2	3	PO	THF	60	60	3	100	93	98:2	31	this work
3	3	PO	THF	60	60	20	1000	34^d^	>99:1	17	this work
4	3	PO	DME	60	60	20	1000	41^d^	>99:1	21	this work
5	3	PO	DME	60	60	20	300	96	>99:1	14	this work
6	[bpy-CTF-Al(OTf)_2_]^+^ [Co(CO)_4_]^−^	PO	DME	50	60	24	30	99	90:10	1.1	^24^
7	Co(CO)_4_^−^ ⊂ Cr-MIL-101	EH^e^	DME	60	60	1	200	88	NA	176	^15^
8	[PPN]^+^[Co(CO)_4_]^−^ BF_3_·Et_2_O	PO	DME	80	62	24	100	77	NA	3.2	^9^
9	[Cp_2_Ti(THF)_2_]^+^ [Co(CO)_4_]^−^	PO	DME	60	62	4	20	95	NA	4.8	^10^
10	[(salph)Al(THF)_2_]^+^ [Co(CO)_4_]^−^	PO	neat	50	62	1	100	95	NA	95	^11^
11	[(salph)Cr(THF)_2_]^+^ [Co(CO)_4_]^−^	PO	DME	22	1	6	50	98	96:4	8.2	^21^

^a^Epoxide/Co: feed molar ratio of epoxide to cobalt species, where the cobalt content of the catalyst was determined from ICP. ^b^Yield of lactone and product ratios determined by ^1^H NMR spectroscopy with an internal standard. ^c^Site time yield: moles of lactone generation per mole of cobalt in catalyst per hour within the reaction time *t*. ^d^Remainder was unreacted PO. ^e^1,2-epoxyhexane. NA: Not analysis.

To establish the activity is unique to [Cr-metalated porous porphyrin polymer]^+^[Co(CO)_4_]^−^, polymer **1**, complex **2**, and K[Co(CO)_4_] were individually tested for PO carbonylation. These systems (10 mg) showed negligible lactone formation when subjected to a 3 h at 60 °C with PO (1.5 g, 0.2 M in THF) under 60 bar of CO.

The catalytic activity of catalyst **3** was also tested for PO carbonylation under relatively high ratio PO/catalyst (1000:1). The STY for β-butyrolactone was found to be 17 h^−1^ (Table 1-3). When the reaction was run in a relatively weak coordinating solvent, such as dimethoxylethane (DME), the STY improved to 21 h^−1^ (Table 1-4).

To easily separate the product from the residual PO, the feed ratio of PO/catalyst was reduced to 300:1 and the reaction was allowed to proceed in DME for 20 h. We found that the yield of β-butyrolactone was 96% and the acetone content was less than 1%. This suggests that the acetone content for catalyst **3** was far less than for our previous heterogeneous catalyst (10%) (Table 1-6).

To evaluate the reusability of catalyst **3**, the catalyst was separated from the reaction solution by simple precipitation and decantation, and it was then dried for the next cycle of catalysis. The catalytic performance of the recovered catalyst is shown in Table 2. Compared with the first cycle, the ratio of β-butyrolactone to acetone in the fourth run was maintained at 94:6; however, the yield of β-butyrolactone was reduced from 95% to 90%, an 5% reduction. To identify the possible reason for the activity loss through recycling, ICP was used to examine the recovered catalyst (after four cycles). It was found that the Cr content was retained (3.5 wt%) and the Co content was reduced, from 1.9 wt% to 1.3 wt%. This result suggests that the reduced activity of catalyst **3** after successive runs can be ascribed to the leaching of Co(CO)_4_^−^.

**Table 2:** Recyclability of catalyst **3** for PO carbonylation^a^.

Cycle	Yield (%)	β-butyrolactone:Acetone^b^
1	95	98:2
2	94	97:3
3	95	97:3
4	90^c^	94:6
5^d^	95	>99:1

^a^Reaction condition: catalyst 30 mg, THF 6.0 g, the feed molar ratio of PO/cobalt 100, CO (60 bar), 60 °C, 3 h. ^b^Determined by ^1^H NMR spectroscopy. ^c^5% unreacted PO. ^d^PO carbonylation using the regenerated catalyst.

The heterogeneous nature of catalysis by the Cr-metalated porphyrin polymer can be further demonstrated by testing the activity of PO carbonylation using the decanted liquid fraction after catalyst precipitation. Specifically, the catalyst **3** (10 mg) was heated in THF (1.7 g) at 60 °C for 3 h under 60 bar of CO, and the resulting mixture was decanted in a glove box. The decanted liquid showed no lactone formation (60 °C, 60 bar CO, 3 h), indicating an negligible Cr species in the decanted liquid for PO carbonylation.

To recover the catalytic performance of the catalyst, the catalyst was treated with KCo(CO)_4_ after four cycles, and its activity was then tested. As expected based on our previous results^24^, the regenerated catalyst showed sufficient activity for PO carbonylation (95% yield). Unexpectedly, the side-product acetone was totally suppressed, leading to a high selectivity for lactone. The suppression of acetone formation may be attributed to the removal of impurities during the regeneration of the catalyst. These results indicate that catalyst **3** exhibited not only high catalytic activity but also outstanding recyclability and selectivity, which supports further study of this heterogeneous catalyst for industrial applications.

## Conclusion

In summary, a novel heterogeneous catalyst, [Cr-metalated porous porphyrin polymer]^+^[Co(CO)_4_]^−^, was rationally designed and synthesized. The synthetic procedure involves the reaction of CrCl_2_ with porous porphyrin polymer and subsequent substitution of Cl^−^ with Co(CO)_4_^−^. The catalytic performance of this heterogeneous catalyst was evaluated using PO carbonylation. The activity and selectivity of the heterogeneous catalyst was superior to our previous heterogeneous catalyst and comparable to that of various homogeneous catalysts. Moreover, the catalyst was able to be recycled by precipitation and decantation for several runs. The results indicate that this Cr-porphyrin-based heterogeneous catalyst is a promising candidate for sustainable epoxide carbonylation in industry

## Methods

### Materials

All compounds were purchased from Sigma-Aldrich Co. PO was refluxed over a mixture of KOH/CaH_2_, and distilled under a nitrogen atmosphere prior to use. THF and DME were distilled with sodium/benzophenone under nitrogen.

## Experimental Procedures

### Synthesis of polymer 1

It was synthesized according to Xiao’s reported procedure^25^.

### Synthesis of complex 2

Polymer **1** (500 mg, 0.69 mmol repeat unit, reddish) was added to DMF (60 mL). The solution was stirred at 130 °C for 4 h. Then, the reaction temperature was increased to 170 °C, and CrCl_2_ (170 mg, 1.38 mmol, 2.0 equ.) was added to the solution. After 16 h, another batch of CrCl_2_ (170 mg) was added to the solution, refluxing at 170 °C for 44 h. The reaction mixture was cooled to room temperature and poured into ice-cold water (300 mL). After the solid was filtered out and washed with excess water and THF, it was dried under vacuum at 120 °C for 32 h, yielding 465 mg of dark product^26^.

### Synthesis of catalyst 3

In a glove box, KCo(CO)_4_ (1.63 g), complex **2** (0.40 g), and THF (10 mL) were mixed in a vial. After stirring at room temperature for 36 h, the solution was filtered and washed several times with THF. THF was added into a flask containing the catalyst. The solution was stirred for several hours, filtered and washed with THF. The catalyst was purified several times using the process of dispersion/filtration/washing with THF. The product (0.38 g) was obtained after drying under vacuum^24^.

### PO carbonylation

In a typical reaction, to a stainless steel reactor (100 mL) was added catalyst (10 mg), PO solution (0.573 g, 0.5 M in THF), and THF (1.427 g) in a glove box. The reactor was purged with ~2 bar of CO and filled with 60 bar of CO. The reaction was run at 60 °C for 3 h. After cooling the reactor with an ice bath and slowly releasing excess gas, the reaction solution was transferred to a vial and recorded the weight. The crude product was filtered through the celite, weighed, and characterized using ^1^H NMR spectrum with naphthalene as an internal standard. Notably, in the recycle, 30 mg of catalyst was used, meanwhile, PO and THF amount were also amplified three times. Poor solvent (pentane/THF 2:1) was used to assist the precipitation of catalyst^24^.

### Characterization

The morphology and composition of the samples were characterized using a Hitachi Model S-4800 FESEM system and EDX. The BET surface area were conducted at 77 K. The IR spectra were recorded on a Shimadzu IRAffinity-1 FT-IR spectrometer using KBr pellets. ^1^H NMR spectra were recorded in CDCl_3_ using a Bruker advance IV (400 MHz).

## Electronic supplementary material

Supplementary Information
