# Supplementary material for: A Metalated Porous Porphyrin Polymer with [Co(CO)4]− Anion as an Efficient Heterogeneous Catalyst for Ring Expanding Carbonylation
Source: Sci Rep. 2018 Sep 5;8:13243. doi: 10.1038/s41598-018-31475-6 (PMC6125460; doi:10.1038/s41598-018-31475-6)
Supplement: Supplementary file 1 — Supplementary Information [file 41598_2018_31475_MOESM1_ESM.pdf]

A Metalated Porous Porphyrin Polymer with $[Co(CO)_4]^-$ Anion as an Efficient Heterogeneous Catalyst for Ring Expanding Carbonylation

*Jianwei Jiang and Sungho Yoon**

Supporting Information

Materials and methods: All compounds were purchased from Sigma–Aldrich and used without further purification. The morphology and composition of the samples were characterized using a Hitachi Model S-4800 FESEM system and EDX. The BET surface area were conducted at 77 K. The IR spectra were recorded on a Shimadzu IRAffinity-1 FT-IR spectrometer using KBr pellets. ^{1}H NMR spectra were recorded in $CDCl_3$ using a Bruker advance IV (400 MHz).

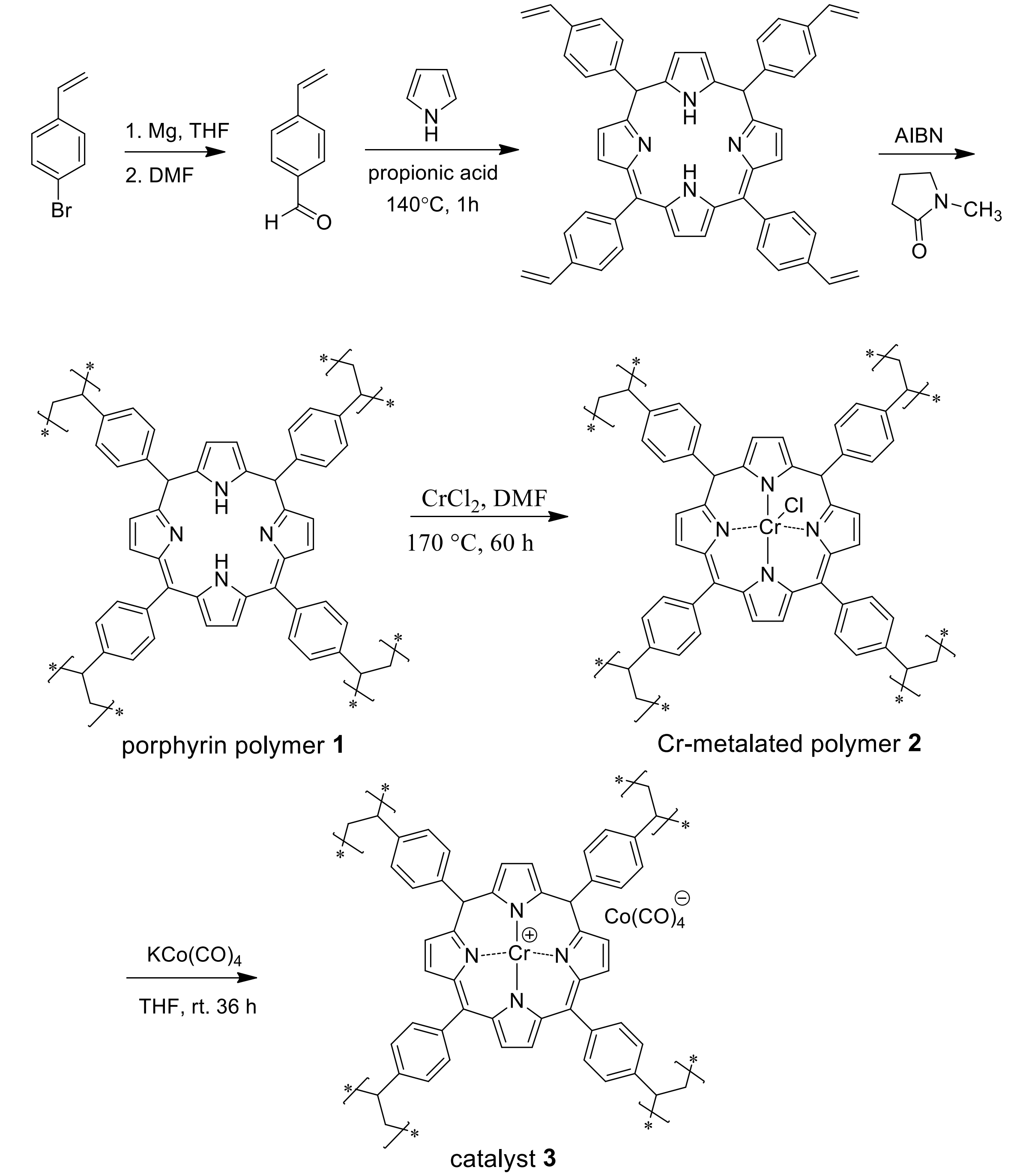

Figure S1. Synthesis of catalyst **3**.

Synthesis of 4-vinylbenzaldehyde[1]

Firstly, the Grignard reagent was prepared from 4-bromostyrene (16.03 g, 87.4 mmol) and Mg (2.56 g, 106.7 mmol) in THF (160 mL). After the solution was cooled to 0 °C, DMF (20 mL) was added under N_2, and stirred at room temperature for 15 h. The reaction mixture was quenched by saturated NH_4Cl solution (80 mL), extracted with ethyl acetate. The organic phase was washed with brine, dried over $MgSO_4$, filtered, concentrated under vacuum, and purified by column chromatography on silica gel (petroleum ether/EtOAc (v/v): 20/1). The 4-vinylbenzaldehyde as yellowish oil (7.54g, 65% yield) was finally obtained. Notably, room temperature was chosen for drying the product, because we observed the polymerization of 4-vinylbenzaldehyde at 40 °C during the vacuum drying process.

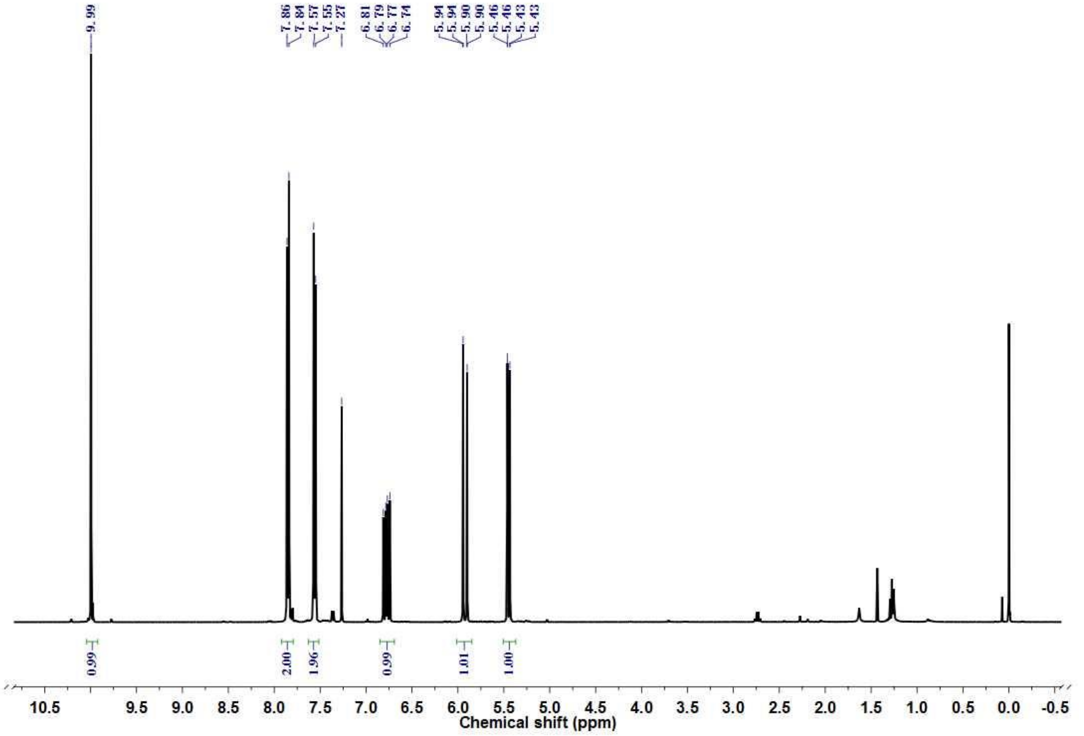

^{1}H NMR spectrum of 4-vinylbenzaldehyde recorded in $CDCl_3$.

Synthesis of tetrastyrylporphyrin monomer[1]

In a 500 mL flask, propionic acid (340 mL) was preheated to 140 ˚C in air, then, pyrrole (2.4 mL, 34.3 mmol) and 4-vinylbenzaldehyde (4.52 g, 34.3 mmol) were added. After 1 h, the solution was cooled to room temperature, filtered, and washed with methanol and ethyl acetate. The product was obtained as purple crystals (3.70 g, 15 % yield) after vacuum at room temperature for 36 h.

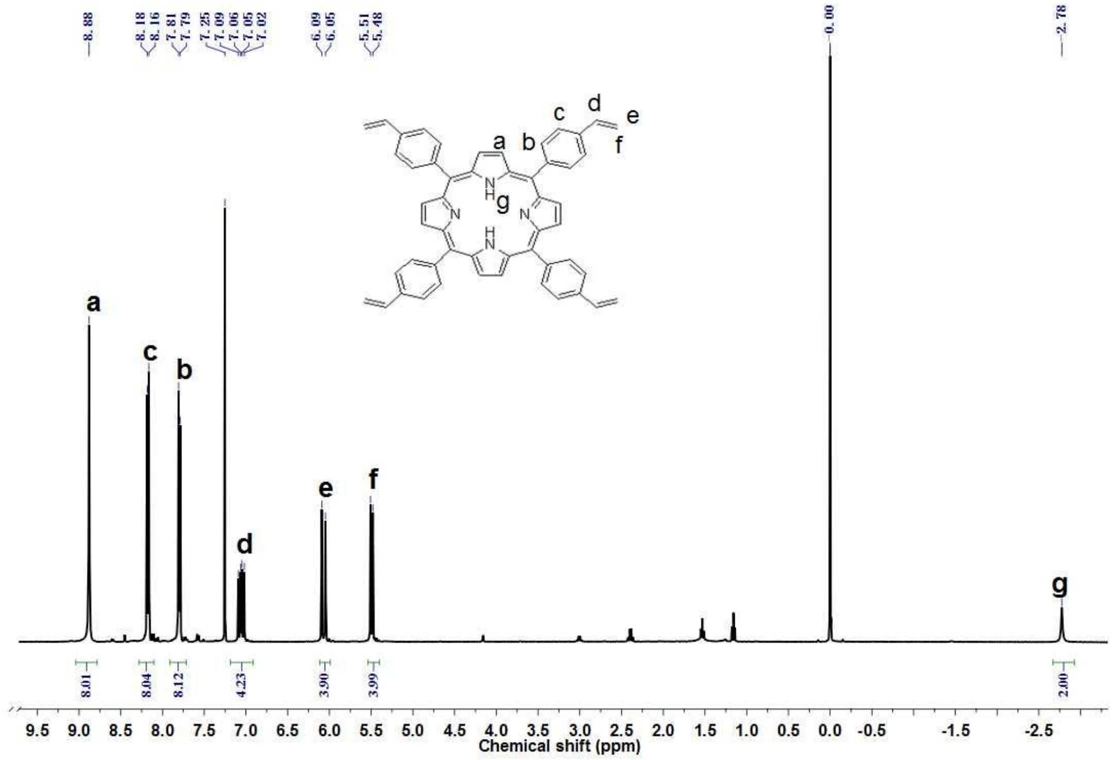

^{1}H NMR spectrum of tetrastyrylporphyrin monomer recorded in $CDCl_3$.

Synthesis of polymer 1[1]

The porous organic porphyrin polymer **1** was synthesized from a solvothermal polymerization of the monomer. Monomer (1.89 g) was dissolved in N-methyl-2-pyrrolidone (20 mL), followed by the addition of AIBN (95 mg). Then, the autoclave was kept at 200 °C for 72 h. The product (reddish brown color) was obtained after washing with DMF and purifying by Soxhlet extraction (CH_2Cl_2, 72 h; 1.72 g, 90% yield).

Synthesis of complex 2[2]

Polymer **1** (500 mg, 0.69 mmol repeat unit, reddish) was added to DMF (60 mL). The solution was stirred at 130 °C for 4 h. Then, the reaction temperature was increased to 170 °C, and $CrCl_2$ (170 mg, 1.38 mmol, 2.0 equ.) was added to the solution. After 16 h, another batch of $CrCl_2$ (170 mg) was added to the solution, refluxing at 170 °C for 44 h. The reaction mixture was cooled to room temperature and poured into ice-cold water (300 mL). After the solid was filtered out and washed with excess water and THF, it was dried under vacuum at 120 °C for 32 h, yielding 465 mg of dark product.

Synthesis of catalyst 3[3]

In a glove box, $KCo(CO)_4$ (1.63 g), complex **2** (0.40 g), and THF (10 mL) were mixed in a vial. After stirring at room temperature for 36 h, the solution was filtered and washed several times with THF. THF was added into a flask containing the catalyst solid. The solution was stirred for several hours, filtered and washed with THF. The catalyst was purified several times using the process of dispersion/filtration/washing with THF. The product (0.38 g) was obtained after drying under vacuum.

PO carbonylation[3]

In a typical reaction, to a stainless steel reactor (100 mL) was added catalyst (10 mg), PO solution (0.573 g, 0.5 M in THF), and THF (1.427 g) in a glove box. The reactor was purged with ~2 bar of CO and filled with 60 bar of CO. The reaction was run at 60 °C for 3 h. After cooling the reactor with an ice bath and slowly releasing excess gas, the reaction solution was transferred to a vial and recorded the weight. The crude product was filtered through the celite, weighed, and characterized using ^1H NMR spectrum with naphthalene as an internal standard. Notably, in the recycle, 30 mg of catalyst was used, meanwhile, PO and THF amount were also amplified three times. Poor solvent (pentane/THF 2:1) was used to assist the precipitation of catalyst.

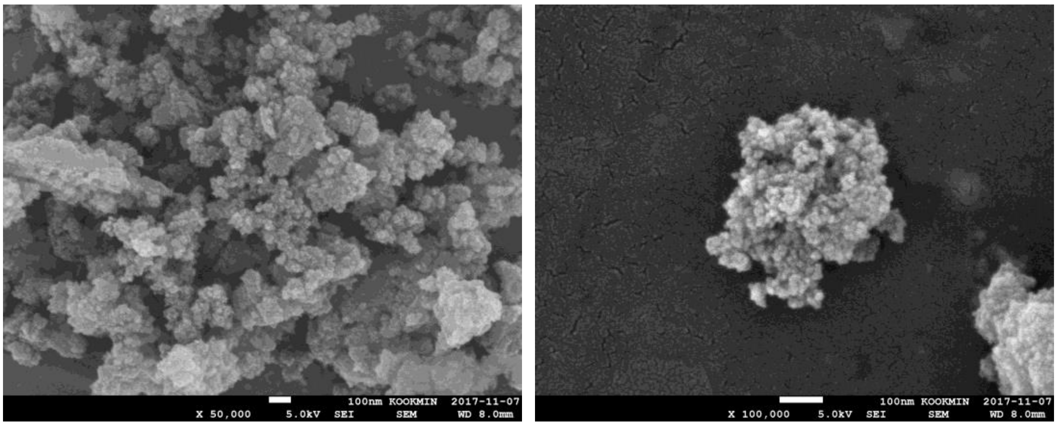

Figure S2. SEM images of polymer **1**.

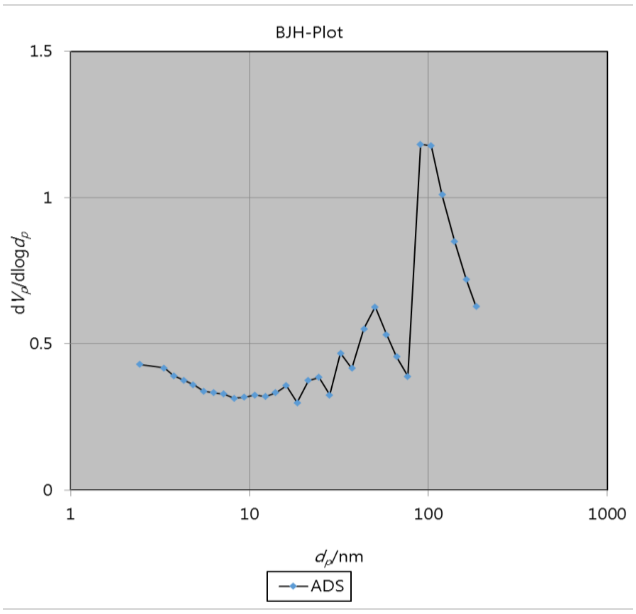

Figure S3. BJH pore-size distribution for polymer **1**.

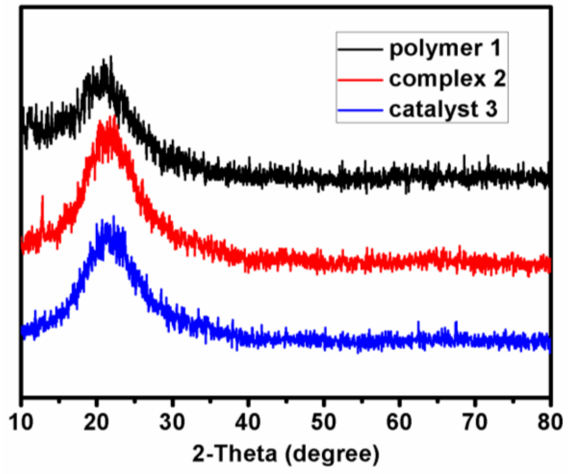

Figure S4. XRD patterns of the polymer **1**, complex **2** and catalyst **3**.

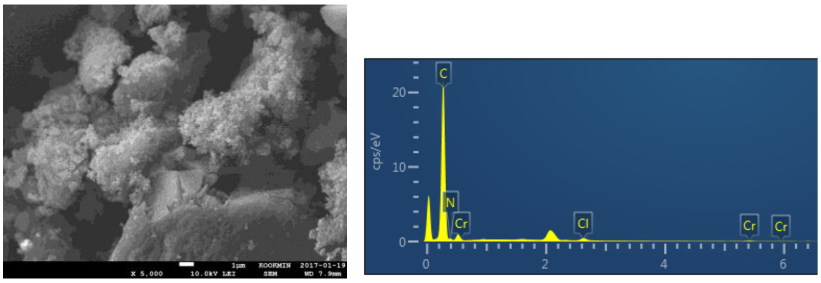

Elem.	Wt%
C	84.35
**N**	**12.11**
Cl	1.27
**Cr**	**2.27**
Total:	100.00

Figure S5. SEM and EDS of complex **2** synthesized from $CrCl_2$ and polymer **1** in DMF for 1 h.

the atomic ratio of Cr/N: $\frac{2.27}{52} / \frac{12.11}{14} = 0.05$

Theoretically, the ratio of Cr/N: 1/4 = 0.25

Therefore, the proportion of Cr metalated porphyrin in the polymer **1**:

$$\frac{0.05}{0.25} \times 100\% = 20\%$$

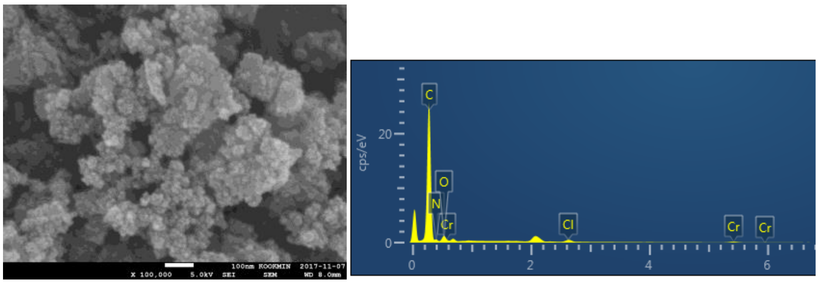

Elem.	Wt%
C	87.18
N	7.45
Cl	1.53
Cr	3.84
Total:	100.00

Figure S6. SEM image and EDX of complex **2** synthesized from $CrCl_2$ and polymer **1** in DMF for 59 h.

the atomic ratio of Cr/N: $\frac{3.84}{52} / \frac{7.45}{14} = 0.1387$

Theoretically, the ratio of Cr/N: 1/4 = 0.25

Therefore, the proportion of Cr metalated porphyrin in the polymer **1**:

$$\frac{0.1387}{0.25} \times 100\% = 55.5\%$$

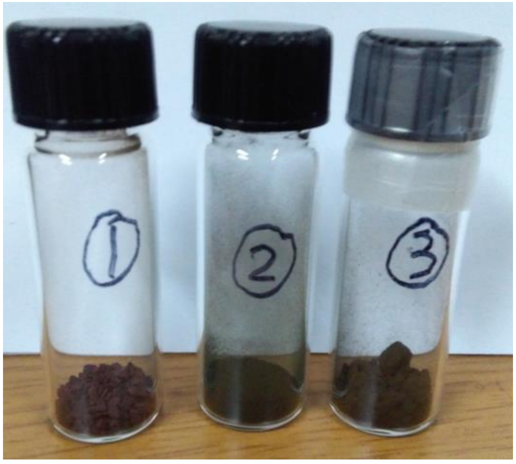

Figure S7. Polymer **1**, complex **2** and catalyst **3**.

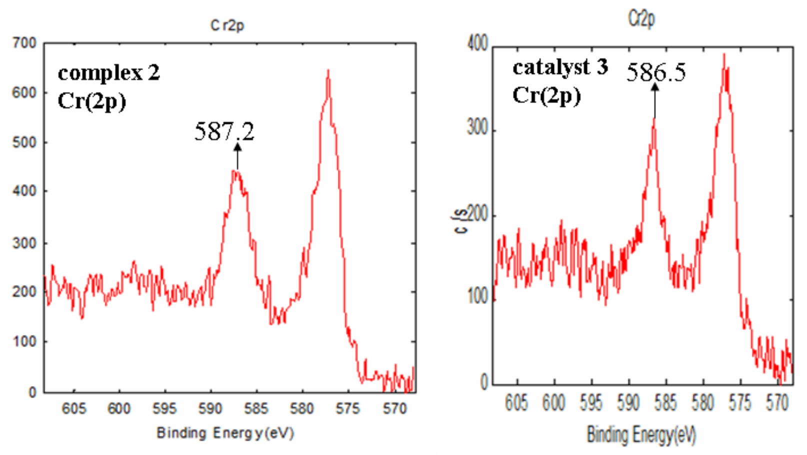

Figure S8. XPS of Cr(2p) of complex **2** and catalyst **3**.

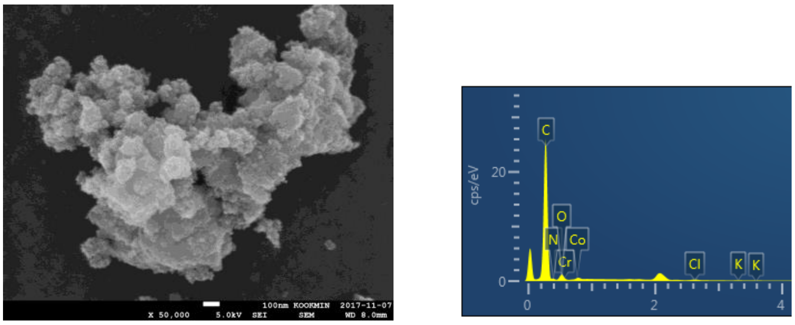

Elem.	Wt%	Atomic%
C	80.96	87.13
N	7.51	6.93
O	5.47	4.42
Cl	0.46	0.17
K	0.17	0.06
Cr	3.53	0.88
Co	1.90	0.42
Total:	100.00	

Figure S9. SEM image and EDX of catalyst **3**.

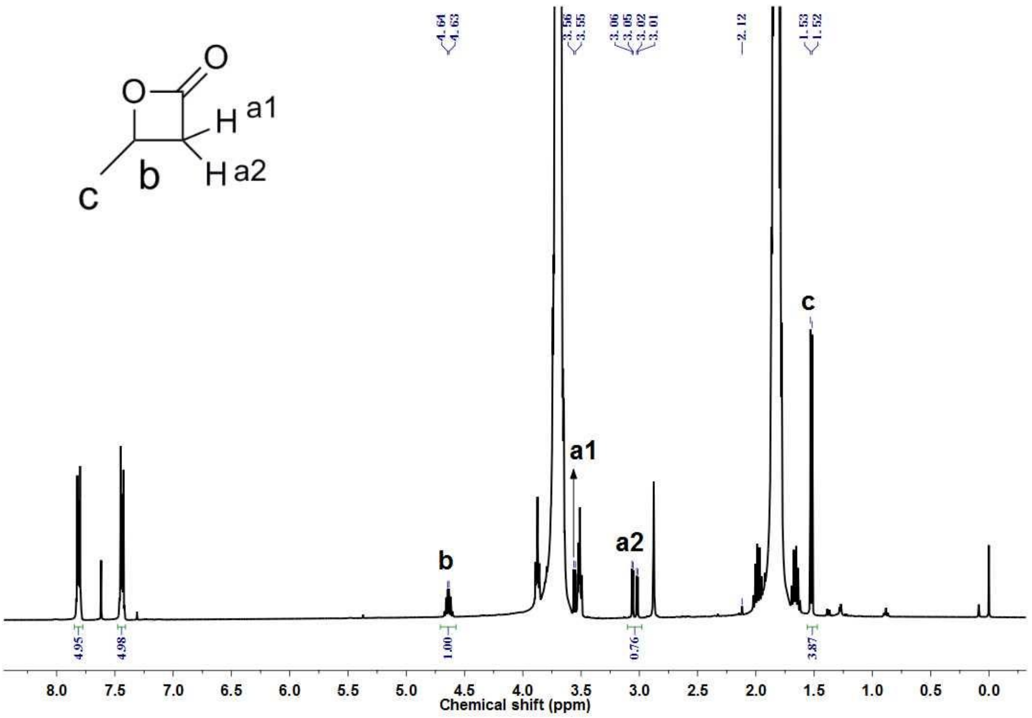

Figure S10. ^{1}H NMR spectrum of the product from PO carbonylation using homogeneous catalyst $[(TPP)Cr(THF)_2]^+[Co(CO)_4]^-$ (Table 1-1).

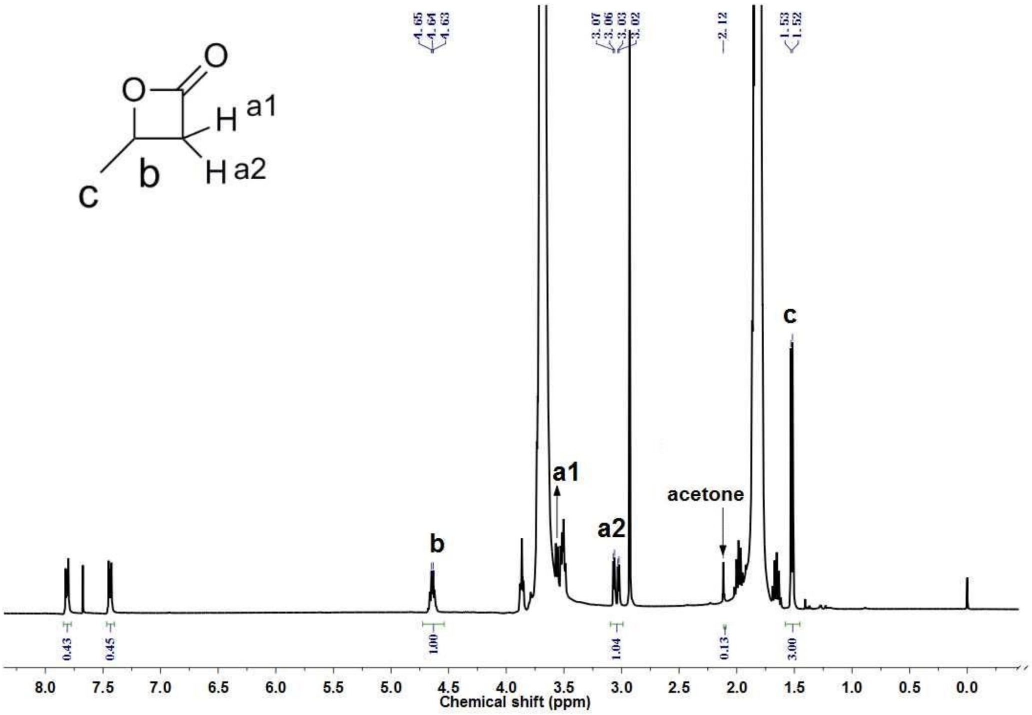

Figure S11. ^{1}H NMR spectrum of the product from PO carbonylation using catalyst **3** (Table 1-2).

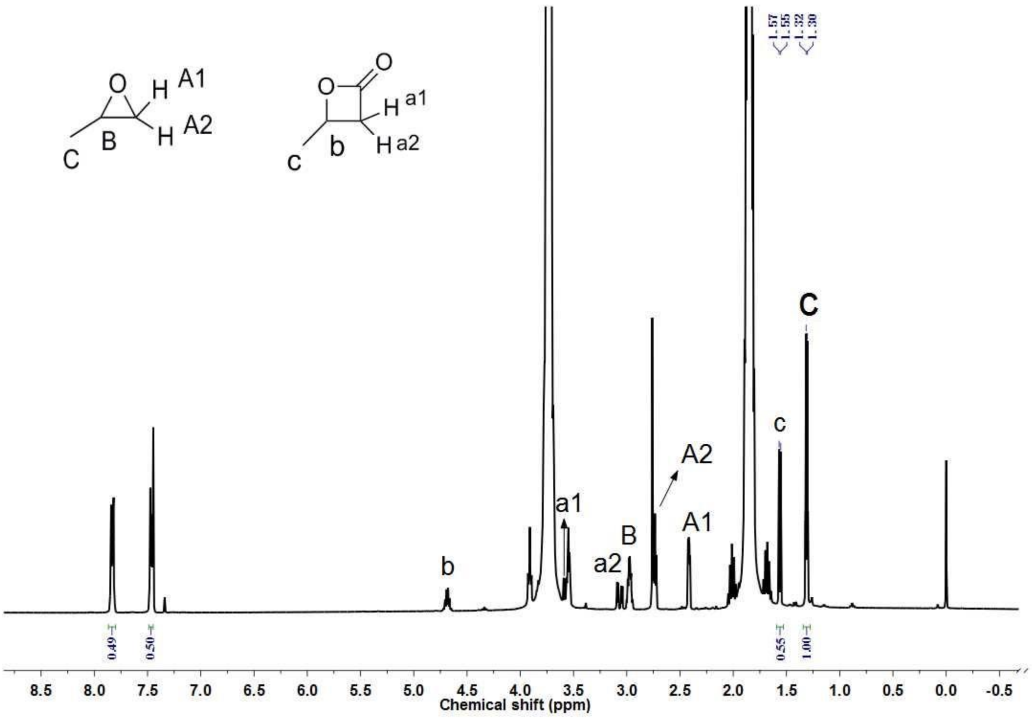

Figure S12. ^{1}H NMR spectrum of the product from PO carbonylation using catalyst **3** (Table 1-3).

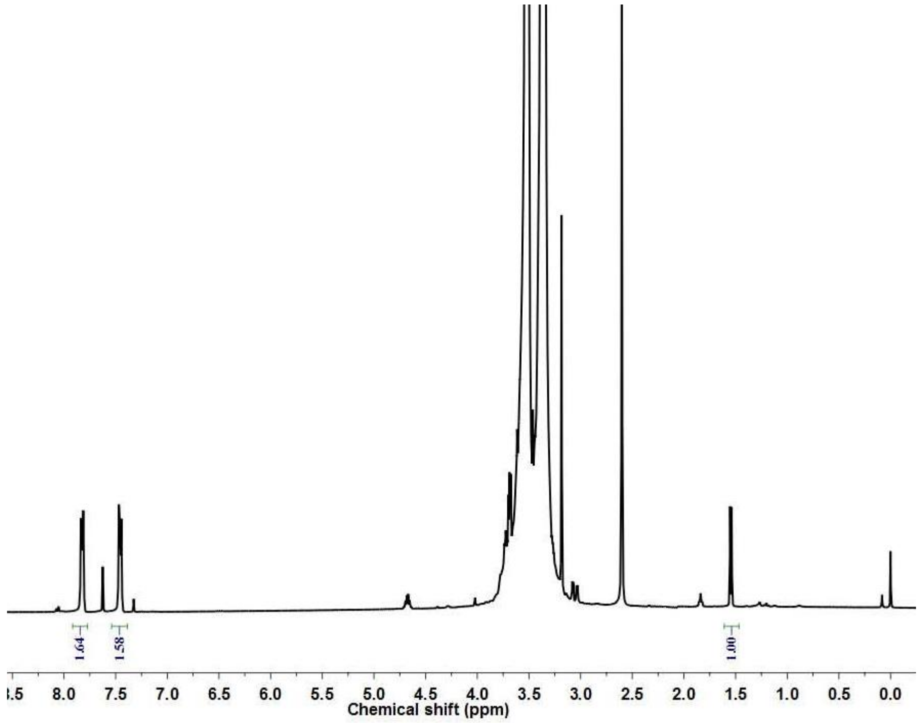

Figure S13. ^{1}H NMR spectrum of the product from PO carbonylation using regenerated catalyst (Table 2-5).

Reference

1. Dai, Z. *et al.* Metalated Porous Porphyrin Polymers as Efficient Heterogeneous Catalysts for Cycloaddition of Epoxides with CO_2 under Ambient Conditions. *J. Catal.* **338,** 202-209 (2016).
2. Chen, P., Chisholm, M. H., Gallucci, J. C., Zhang, X. & Zhou, Z. Binding of Propylene Oxide to Porphyrin− and Salen−M(III) Cations, Where M = Al, Ga, Cr, and Co. *Inorg. Chem.* **44,** 2588-2595 (2005).
3. Rajendiran, S., Natarajan, P. & Yoon, S. A Covalent Triazine Framework-based Heterogenized Al-Co Bimetallic Catalyst for the Ring-expansion Carbonylation of Epoxide to β-lactone. *RSC Adv.* **7,** 4635-4638 (2017).
